# The Efficacy of Tai Chi and Qigong Exercises on Blood Pressure and Blood Levels of Nitric Oxide and Endothelin-1 in Patients with Essential Hypertension: A Systematic Review and Meta-Analysis of Randomized Controlled Trials

**DOI:** 10.1155/2020/3267971

**Published:** 2020-07-30

**Authors:** Dingcheng Liu, Lan Yi, Meixiao Sheng, Gang Wang, Yanqin Zou

**Affiliations:** ^1^The First College of Clinical Medicine, Nanjing University of Chinese Medicine, Nanjing, Jiangsu, China; ^2^Affiliated Hospital of Nanjing University of Chinese Medicine, Nanjing, Jiangsu, China; ^3^Nanjing Boda Kidney Hospital Affiliated to Nanjing University of Chinese Medicine, Nanjing, Jiangsu, China

## Abstract

**Objective:**

Tai Chi and Qigong are the two similar traditional Chinese wellness exercises. A strong body of published clinical randomized controlled trials (RCTs) has investigated the health benefits of Tai Chi and Qigong exercises (TCQE) in patients with essential hypertension (EH). This is the first meta-analysis to evaluate the efficacy of TCQE on blood pressure (BP) and blood levels of nitric oxide (NO) and endothelin-1 (ET-1) in EH patients and explore the potential antihypertensive mechanism of TCQE.

**Methods:**

We conducted a literature retrieval for Chinese and English studies in seven databases from their respective inceptions until January 14, 2020. All RCTs examining clinical efficacy of TCQE for EH patients were considered. The major therapeutic outcomes of TCQE were changes in the blood levels of NO, ET-1, and BP in EH patients. Methodological quality of the included RCTs was detected via The Cochrane Risk of Bias tool. We evaluated the data reported and performed the meta-analysis by Review Manager 5.3 software.

**Results:**

9 RCTs involving 516 EH patients were included. The intervention duration lasted from 1.5 months to 6 months. The results of comprehensive analysis showed that compared with control interventions, experimental interventions were more effective in reducing the systolic blood pressure and the diastolic blood pressure and contributed higher blood levels of NO and lower blood levels of ET-1.

**Conclusions:**

TCQE could be an effective complementary and alternative therapy for EH. The lower BP in EH patients who practice TCQE may have some connection with exercise-related increased blood NO levels and decreased blood ET-1 levels. However, further research is needed to make clear the efficacy of TCQE in management of EH and the mechanism of lowering BP in TCQE.

## 1. Background

### 1.1. Description of the Condition

Hypertension (HT) is a major health care problem, affecting more than 1 billion people globally and being a major risk factor for stroke, chronic kidney disease, and myocardial infarction [1, 2]. Almost 26% of the adult population experiences HT, while its prevalence may rise to 29% by the year 2025 [3]. HT is a multifactorial disease involving environmental and genetic factors together with risk-conferring behaviors. The cause of the disease is identified in 10% of the cases (secondary hypertension), but in 90% of the cases no etiology is found (primary or essential hypertension [EH]) [4].

Endothelial dysfunction (ED) may be both a cause and a consequence of HT [5]. Although the endothelium has a number of important functions, the term ED is commonly used to describe impairment in its vasodilatory capacity. It is increasingly recognised that there is a strong bidirectional association between EH and ED [5, 6]. In addition, ED emerges as a promising therapeutic target of agents that are readily available in clinical practice. In this context, a better understanding of its role in EH becomes of great importance [7].

The healthy endothelium has a naturally vasodilated resting state, mainly due to the action of nitric oxide (NO) [5]. NO is the main vasorelaxing factor produced by endothelial cells that acts to maintain vascular tone. Reduced bioavailability of NO appears to be the key process through which ED is manifested in HT [8, 9].

Endothelin-1 (ET-1) was identified in 1988 following a lengthy, intensive search for the elusive endothelial cell-derived constricting factor. This discovery came shortly after the description of NO as the endothelial-derived relaxing factor, generating high hopes that many vascular diseases, foremost of which was HT, would be more effectively treated by restoring the normal balance of these opposing vasoactive agents [10]. ET-1 is an important circulatory indicator of ED and the most potent endogenous vasoconstrictor, exerting a wide range of biologic effects that can influence systemic blood pressure (BP) and increase the risk of HT development [10–13]. Elevated plasma level of ET-1 has been observed in patients with EH [14]. There is a significant association between high plasma ET-1 level and development of HT in a general population [15].

### 1.2. Description of the Intervention

Despite the fact that during the last decades, several antihypertensive drug therapies have been introduced and tested in clinical trials, as both monotherapies and combination therapies, EH constitutes a serious burden on public health because there is no definitive cure for the disease. The majority of hypertensive patients require long-term treatment. However, this can lead to patients being permanently dependent on medications and susceptible to long-term cardiovascular complications. Effective treatment of EH is limited by availability, cost, and adverse effects of conventional western medicine treatment [16–18]. Due to the limitations and concerns with current available HT treatments, many hypertensive patients, especially in Asia, have turned to traditional Chinese medicine (TCM). It appeared that TCM was effective for HT in clinical use, such as Qigong and Tai Chi [19].

Qigong exercise is Chinese health exercises that have been used and developed for thousands of years to optimize energy within the body, mind, and spirit. It can affect many functions of the body and be beneficial for various “civilization” diseases such as HT [20–22]. Tai Chi has its roots in China and is based on the Tao (balance of Yin/Yang) philosophy to improve health. Over the years Tai Chi has become more focused on health promotion compared to martial art. It is becoming more commonplace as a preventative and rehabilitative therapeutic tool within the Western medical community, especially its use with the most rapidly increasing segment of the population—elders [23–25]. Obviously, Qigong exercise and Tai Chi have similar characteristics and the same advantages. Tai Chi and Qigong exercises (TCQE) require no special equipment and can be practised almost anytime and anywhere, indoors or outdoors, and either in a group or alone. Moreover, TCQE are a low-cost exercise, and they can be easily implemented in the community [24, 26]. Last but not least, Tai Chi has been widely practised in China for centuries by people of all ages and both sexes [23]. There were no studies that found Tai Chi worsened a condition. A recent systematic review on the safety of Tai Chi found adverse events were typically minor and primarily musculoskeletal; no intervention-related serious adverse events have been reported [27].

### 1.3. How the Intervention Might Work

In older individuals, Tai Chi training could further lower systolic BP from 172 to 159 mmHg for patients who had a 12-minute walk [28]. In patients with HT, studies showed that Tai Chi training might decrease systolic BP (range: −7 to −32 mmHg) and diastolic BP (−2.4 to−18 mmHg) [29]. The underlying mechanisms explaining the effects of Tai Chi remain largely unclear [30]. Whether the BP decrease is a consequence of the mental or physical relaxation aspects of the exercise is still under investigation [31].

Previous studies have demonstrated that Qigong is beneficial in lowering HT as well as improving life qualities [32]. For hypertensive patients, combining qigong practice with drug therapy for hypertensive patients resulted in reduced incidence of stroke and mortality and reduced dosage of drugs required for BP maintenance [33]. Breathing exercise guided by the BIM (Breathe with Interactive Music) device for 10 min daily is an effective nonpharmacological modality to reduce BP. It is not known if qigong lowers BP by this mechanism [34, 35].

According to the available literature, TCQE may change the blood levels of endothelial factors (NO and ET-1) that are involved in regulation of vascular endothelial function, which may be the potential mechanism of TCQE reducing BP in EH patients. ED is associated with lack of exercise. Regular practice of Tai Chi may enhance endothelium-dependent dilation in skin vasculature of older individuals [36]. Elderly Tai Chi practitioners have been shown to have higher plasma levels of NO than sedentary individuals at rest and during exercise [37].

### 1.4. Why It Is Important to Do This Review

The current state of research splinters these TCM-based wellness practices by identifying them with different names and treating them as distinct fields of inquiry, reducing the potential for evaluating health outcomes across Qigong and Tai Chi research [38]. Qigong and Tai Chi are both based on theoretical principles that are inherent to TCM [39]. There are foundational similarities between Qigong and Tai Chi intervention protocols, as traditional Tai Chi is typically modified and adapted for ease of dissemination to more closely resemble forms of Qigong. Qigong and Tai Chi are close relatives, having shared theoretical roots, common operational components, and similar links to the wellness and health-promoting aspects of TCM [38]. Because of these similar foundations, in our meta-analysis, we considered the research literature for these two forms of traditional Chinese medical exercises as one body of evidence and investigated the benefits of both forms together for patients with EH. Furthermore, as far as we know, previous systematic evaluations of Tai Chi or Qigong in the control of EH [40–43] have overlooked the evaluation of blood NO and ET-1 levels which are closely related to vascular endothelial function in patients with EH. Last but not least, a number of new randomized controlled trials (RCTs) have been recently published. Accordingly, in our article, we update the latest evidence in clinical RCTs and further review the efficacy of traditional Chinese medical exercises (Tai Chi and Qigong) for the EH patients, focusing on the exercise-related changes of blood NO and ET-1 levels. The primary aim of our study is to evaluate the efficacy of TCQE on lowering the BP of patients with EH. What is more, our second purpose is to examine the potential mechanism of reducing BP by investigating the variations of the blood NO and ET-1 levels in experimental groups and control groups.

## 2. Methods

### 2.1. The Selection Strategy of Literature

A search was applied for Chinese and English literatures in 7 databases including Cochrane, PubMed, Embase, Chinese National Knowledge Infrastructure (CNKI), Chinese Science and Technology Journal Database (VIP), Wanfang Data, and Chinese Biomedical Database (CBM) from their establishment through January 14, 2020. We conducted an analogous search strategy in the Cochrane, PubMed, and Embase. There was no limitation when we adjusted and used these search terms in the Chinese databases.  Table 1 presented the search strategies applied for this review.

### 2.2. Eligibility Criteria

#### 2.2.1. Inclusion Criteria

Inclusion criteria (PICO format) were as follows:

1. Participants: aged over 49 years, with no severe complication, with an explicit diagnosis of EH. The diagnostic criteria for EH were defined by the International Society of Hypertension Guidelines for the Management of Hypertension [44]: systolic blood pressure (SBP) ≥140 mmHg, and/or diastolic blood pressure (DBP) ≥90 mmHg, and/or antihypertensive drug use for at least 2 weeks. Studies that confirmed participants with EH but without providing specific diagnostic criteria for HT were also taken into account.
2. Intervention: TCQE alone or plus control regimen as the primary intervention.
3. Control: Any type of control regimens was acceptable, such as no intervention, usual care, and standard antihypertensive therapy and any kind of physical exercises.
4. Outcomes: primary outcomes were pre- and posttreatment variations in SBP, DBP, and blood levels of NO and ET-1. All trials used continuous outcome as the primary measure.
5. Types of studies: only the RCTs were included.

#### 2.2.2. Exclusion Criteria

Exclusion criteria (PICO format) were as follows:

1. Participants: we excluded participants diagnosed with secondary hypertension, such as drug hypertension, secretory hypertension, and renal hypertension and medical history of cardiovascular diseases and obstructive sleep apnea hypopnea syndrome (OSAHS).
2. Intervention and control: we excluded RCTs if regimens in the intervention groups or control groups included other traditional Chinese medicine therapies, such as Chinese herbal medicine, acupuncture, and Tuina; details of regimens in the intervention groups or control groups were ill-defined.
3. Outcomes: the primary outcome data were incomplete.
4. Types of studies: repeat publications, non-RCTs, pseudo-randomization studies, cohort studies, and case-control studies were excluded.

### 2.3. Selection of Studies

Two independent authors (DL and LY) performed the document screening. First, the titles and abstracts of all literature were filtrated for excluding obviously irrelevant articles. Next, the full texts of all remaining papers were screened to identify those that clearly met the inclusion criteria. Any divergence was settled through consultation with a third author (MS).

### 2.4. Data Extraction

Two authors (DL and LY) independently conducted the data extraction using a standardized form which was specially devised before the start of literature search. The collected data are as follows: publication information, sample size, participants' characteristics (age, sex), diagnosis standard of HT, classification of HT, frequency of intervention of TCQE, interventions for experimental groups and control groups, intervention duration, outcome indicators, and dropout. The collected detailed data are presented in Table 2.

### 2.5. Statistical Analysis

We carried out data synthesis and analysis using Review Manager software (Version 5.3, The Cochrane Collaboration, 2014). All continuous outcomes were extracted from the intact data of the original RCTs and expressed as the mean ± standard deviation without conversion. For continuous data, the mean difference (MD) is selected if outcomes are measured in the same way between studies, while the standardized mean difference (SMD) is selected to combine studies that measure the same outcome but use different methods [54]. BP was measured with various methods including 24 h ambulatory BP monitoring, mercury sphygmomanometer, and automated sphygmomanometer. NO concentrations in the blood were determined using the nitrate reductase (NR) method or enzyme linked immunosorbent assay (ELISA). ET-1 concentrations in the blood were detected by radioimmunoassay (RIA) method or enzyme linked immunosorbent assay (ELISA). As the primary outcomes and continuous variables of our meta-analysis, levels of SBP, DBP, NO, and ET-1 were presented as SMD, both with 95% confidence interval (CI). Cochran Q statistic and the *I*^2^ index were applied to evaluate the degree of statistical heterogeneity. *I*^2^ index more than 50% was identified as significant heterogeneity. Due to prominent clinical heterogeneity, we chose a random-effects model to merge data, whereas a fixed-effects model was chosen because of the low heterogeneity. Sensitivity analysis was conducted to check the source of the statistical heterogeneity. The method is that involved RCTs were excluded one by one and the rest of RCTs were accepted to meta-analysis again to inspect for any result of the change before and after the elimination. Because of insufficient studies (less than 10), funnel plot analysis was not performed for checking the potential publication bias.

### 2.6. Methodological Quality

Two authors (DL and LY) independently evaluated the methodological quality of the eligible RCTs as low risk, high risk, or unclear risk, via Cochrane's “Risk of Bias” tool [55] and solved any discrepancy by discussing with the third author (MS). The evaluation results of methodological quality were based on the following 7 criteria: (1) random sequence generation, (2) allocation concealment, (3) blinding for participants and personnel, (4) blinding of outcome assessment, (5) incomplete outcome data, (6) selective reporting, and (7) other bias.

## 3. Results

### 3.1. Search Results

**Figure 1:**
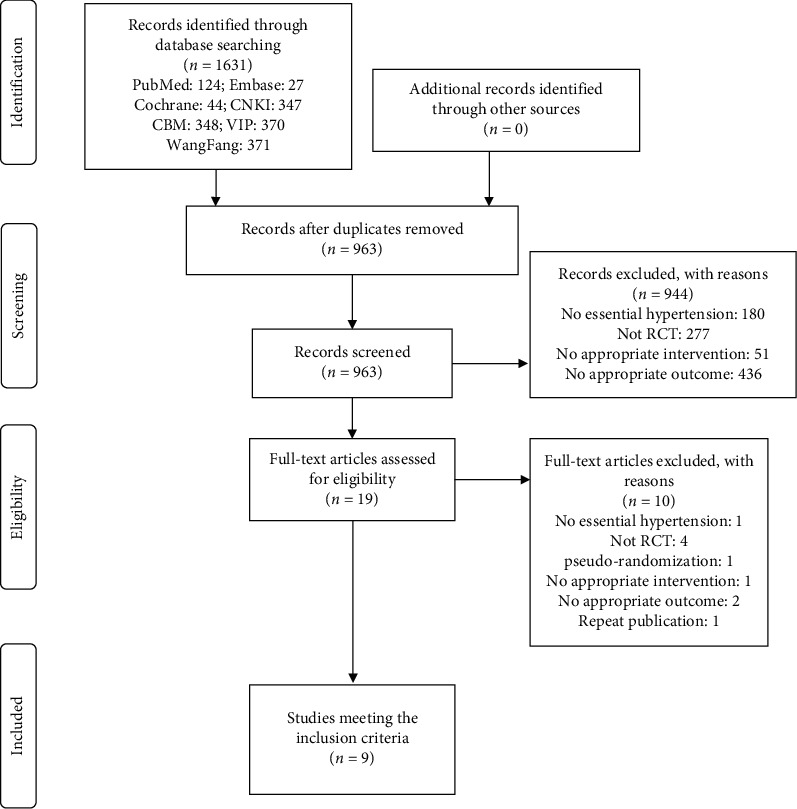
Flowchart of literature screening.

There were 1631 relevant articles retrieved from 7 electronic databases in accordance with the selection strategy of literature. First of all, after excluding 668 repetitive articles in total, we read the title and abstract of the remaining articles, 944 of which were excluded according to eligibility criteria. Then, full texts of the remaining 19 papers were downloaded for further identification, and 10 publications were ruled out based on the inclusion and exclusion criteria. Finally, we brought absolutely eligible 9 RCTs into our meta-analysis. The process of literature screening was shown in Figure 1.

### 3.2. Study Characteristics

#### 3.2.1. Study Design

All the nine qualified RCTs were parallel-designed, of which six were published in journals and three [46, 48, 50] were published as Master's theses. All trials were originated in Chinese single centres between 2006 and 2018, with seven trials published in China and two [45, 51] in American.

#### 3.2.2. Participants

The key characteristics of the included RCTs are summarized in Table 2. In total, there were 516 participants in the nine trials (255 in the experimental groups and 256 in the control groups). The sample sizes ranged from 20 to 42 participants and the average age of subjects ranged from 56 to 70 years. Seven of the studies involved both men and women, and the remaining two [45, 51] did not report the gender of the participants. Seven trials applied diagnostic criteria of Guidelines for Prevention and Treatment of Hypertension in China, of which one [47] used 2004 edition, two [46, 53] used 2005 edition, three [48, 49, 52] used 2010 edition, and one [50] used 2015 edition. Two trials [45, 51] identified participants with EH but did not describe diagnostic criteria. Three studies [48, 50, 53] included patients with EH of stage I and stage II, two [46, 49] included patients with EH of stage I, and one [47] included patients with EH of stage II and stage III, but the remaining three studies [45, 51, 52] did not indicate the stage of EH.

#### 3.2.3. Interventions

Interventions in the experimental groups included different types of Tai Chi (24-movement Yang-style Tai Chi [49, 50, 53], 8-type Tai Chi [52], and Chen-type Tai Chi [47]), and Qigong (Mawangdui Daoyinshu [45], Baduanjin [46, 51], and self-compiled [48]), and the control groups included different regimens of antihypertensive drug (amlodipine besylate or telmisartan alone [46], nifedipine sustained-release tablets [47], and amlodipine besylate [50]), but one study [48] reported only the different types of antihypertensive drugs used, not the dosage of specific drugs. Four studies [46–48, 50] evaluated the effect of Tai Chi or Qigong plus antihypertensive drug versus routine antihypertensive drug, three [45, 51, 53] evaluated the effect of Tai Chi or Qigong alone versus no intervention, one [49] evaluated the effect of Tai Chi versus routine antihypertensive drug, and the other [52] evaluated the effect of Tai Chi plus aerobic exercise (jogging and walking fast) versus aerobic exercise. The intervention duration in the RCTs ranged from 1.5 to 6 months. The frequency of directed TCQE ranged from five to seven days a week, with 30 to 60 minutes a day.

#### 3.2.4. Outcomes

Eight RCTs published the levels of SBP and DBP, nine published the levels of NO in blood, and seven published the levels of ET-1 in blood. All outcome indicators were measured for each subject at baseline and the end of intervention. Adverse events were not recorded in studies. Information about dropouts was reported in three trials [46, 47, 49].

### 3.3. Meta-Analysis

According to dissimilarly clinical interventions in experimental groups and in control groups, we divided the included studies into six subgroups. The first subgroup involving one study [53] compared Tai Chi with no intervention, the second including two studies [45, 51] compared the Qigong with no intervention, the third involving two studies [47, 50] made comparisons between Tai Chi plus antihypertensive drug and antihypertensive drug alone, the fourth including two trials [46, 48] made comparisons between Qigong plus antihypertensive drug and antihypertensive drug alone, the fifth including one trial [49] compared the Tai Chi with antihypertensive drugs, and the sixth involving one study [52] compared the Tai Chi plus aerobic exercise with aerobic exercise alone. Three subgroups (Tai Chi/Qigong vs. no intervention, Tai Chi/Qigong plus antihypertensive drug vs. antihypertensive drug, and Tai Chi plus aerobic exercise vs. aerobic exercise) mainly reflected the efficacy of TCQE for EH patients, while the other subgroup (Tai Chi vs. antihypertensive drug) primarily paid attention to evaluate whether Tai Chi had a better effectiveness for EH patients than antihypertensive drug.

#### 3.3.1. Blood Pressure

Eight trials involving 451 EH patients measured the value of SBP and DBP to assess the efficacy of TCQE in reducing BP.

**Figure 2:**
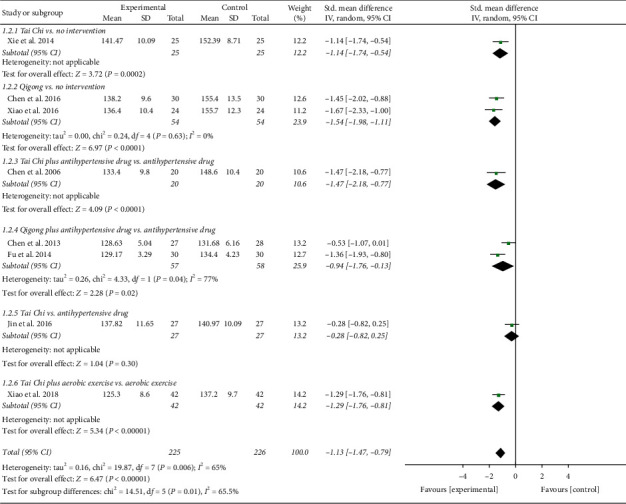
Effects of TCQE on SBP levels in patients with EH.

The result of compositive analysis proved that the SBP of EH patients in the experimental groups were obviously lower than those in the control groups (SMD = −1.13; 95% CI: −1.47 to −0.79; *P* < 0.00001; random-effects model; Figure 2) after interventions, which explained that TCQE were effective in lowering SBP for EH patients. The results of all subgroup analysis except for the fifth subgroup also confirmed this finding. The result of the fifth subgroup showed that there was no significantly statistical difference between Tai Chi and antihypertensive drug in reducing SBP (SMD = −0.28; 95% CI: −0.82 to 0.25; *P*=0.30). The results of combined analysis had distinct heterogeneity (Chi^2^ [chi-square] = 19.87, df [degree of freedom] = 7; *I*^2^ = 65%). We conducted a sensitivity analysis and found that the study of Jin et al. [49] made a great contribution to the heterogeneity. The intervention duration of their trial was just 1.5 months which was obviously shorter than that of the other trials, indicating that the duration of intervention may be a potential source of heterogeneity.

**Figure 3:**
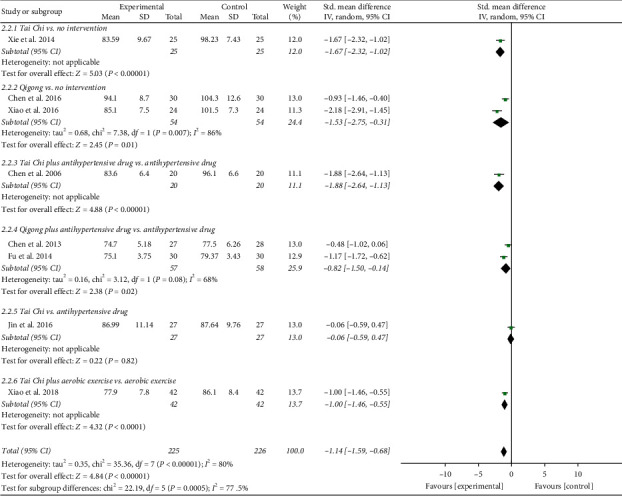
Effects of TCQE on DBP levels in patients with EH.

In addition, the result of combined analysis revealed that, after treatment, experimental plans contributed to a greater drop in DBP of EH patients (SMD = −1.14; 95% CI: −1.59 to −0.68; *P* < 0.00001; random-effects model; Figure 3) compared with the control plans, indicating that TCQE were directly related to the effect of reducing DBP. The analytical results of all subgroups except for the fifth subgroup also confirmed the conclusion, while the results of the fifth subgroup analysis showed no statistically noteworthy difference between Tai Chi and antihypertensive drug in the effect of reducing DBP (SMD = −0.06; 95% CI: −0.59 to 0.47; *P*=0.82). There was obviously statistical heterogeneity in the results of the second subgroup and the comprehensive analysis (comprehensive analysis: Chi^2^ = 35.36, df = 7; *I*^2^ = 80%; the second subgroup: Chi^2^ = 7.38, df = 1; *I*^2^ = 86%). The cause of heterogeneity in the second subgroup may be that the Qigong styles between two studies were not completely consistent. Sensitivity analysis showed that the statistically significant heterogeneity in comprehensive analysis was still mainly derived from the research of Jin et al. [49].

As described, although there was no statistical significance between Tai Chi and antihypertensive drugs in antihypertensive effect, comprehensive result displayed that, after treatment, the overall BP value of the EH participants in the experimental groups was lower than that in the control groups, demonstrating that TCQE could reduce the BP levels of patients with EH.

**Figure 4:**
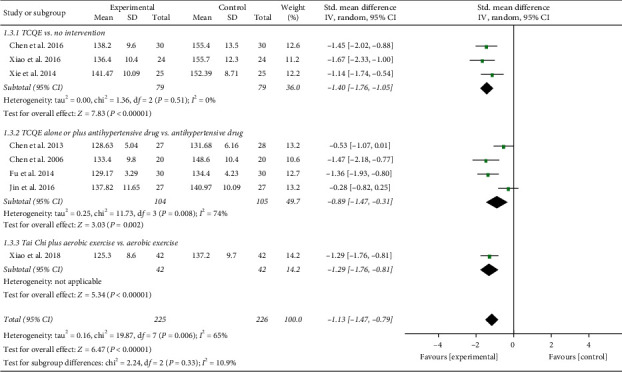
Effects of TCQE on SBP levels (subgroups based on different controls).

**Figure 5:**
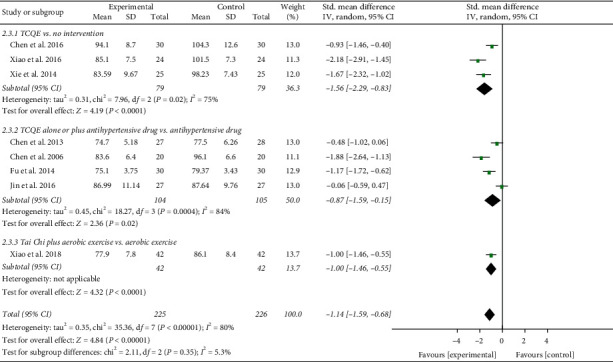
Effects of TCQE on DBP levels (subgroups based on different controls).

We performed further subgroup meta-analysis to investigate the influence of different controls, durations of intervention, or styles of exercises on the BP levels. First, we conducted a subgroup analysis on the basis of different controls. The results showed that TCQE alone or plus control plans had a better efficacy on reducing SBP levels when compared with control plans including no intervention (SMD = −1.40; 95% CI: −1.76 to −1.05; *P* < 0.00001; Figure 4), antihypertensive drug (SMD = −0.89; 95% CI: −1.47 to −0.31; *P*=0.002), or aerobic exercise (SMD = −1.29; 95% CI: −1.76 to −0.81; *P* < 0.00001). Sensitivity analysis showed that the statistically significant heterogeneity (Chi^2^ = 11.73, df = 3; *I*^2^ = 74%) in the second subgroup was mainly due to the study of Jin et al. [49]. Different experimental plans and intervention durations may give rise to the heterogeneity. In addition, compared with control schemes, such as no intervention (SMD = −1.56; 95% CI: −2.29 to −0.83; *P* < 0.0001; Figure 5), antihypertensive drug (SMD = −0.87; 95% CI: −1.59 to −0.15; *P*=0.02), or aerobic exercise (SMD = −1.00; 95% CI: −1.46 to −0.55; *P* < 0.0001), TCQE alone or plus control schemes also made a contribution to a better decrease of DBP levels. There was obviously statistical heterogeneity in the results of the first (Chi^2^ = 7.96, df = 2; *I*^2^ = 75%) and second (Chi^2^ = 18.27, df = 3; *I*^2^ = 84%) subgroups. Sensitivity analysis suggested that the heterogeneity came from the study of Chen [45] and Chen et al. [47], respectively. Different TCQE styles may be the cause of heterogeneity. As described above, TCQE may be effective in lowering BP value.

**Figure 6:**
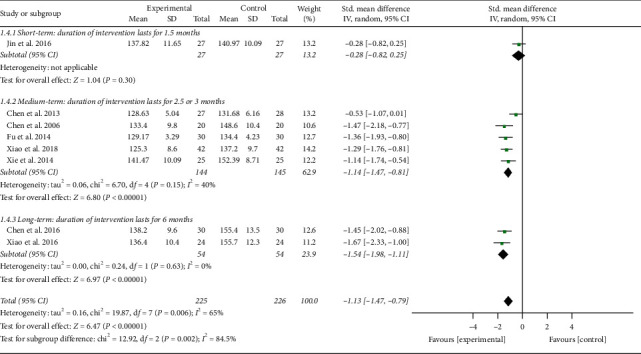
Effects of TCQE on SBP levels (subgroups based on different durations of intervention).

**Figure 7:**
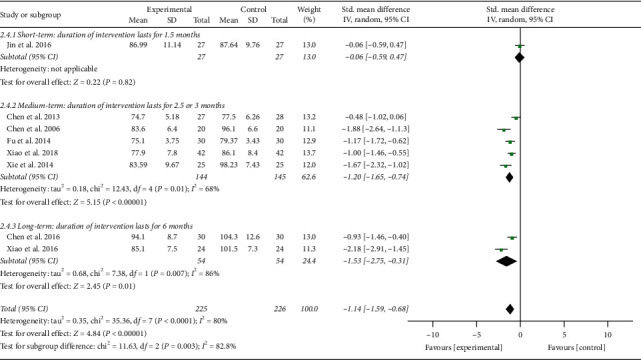
Effects of TCQE on DBP levels (subgroups based on different durations of intervention).

Second, based on different durations of intervention, subgroups were divided into short (1.5 months), medium (2.5 or 3 months), and long (6 months) term. Due to the large *P* value (*P* ≥ 0.05) in the first subgroup, short-term intervention was not statistically significant in changing BP levels. According to the size of SMD value, the efficacy of different intervention duration on SBP was sorted from large to small: long-term (SMD = −1.54; 95% CI: −1.98 to −1.11; *P* < 0.00001; Figure 6) and medium-term (SMD = −1.14; 95% CI: −1.47 to −0.81; *P* < 0.00001). Sensitivity analysis suggested that the statistically moderate heterogeneity (Chi^2^ = 6.70, df = 4; *I*^2^ = 40%) in the second subgroup originated from the study of Chen [46]. Different TCQE styles may be the cause of heterogeneity. Similarly, on the basis of SMD value, the effect of intervention duration on DBP was sorted from large to small: long-term (SMD = −1.53; 95% CI: −2.75 to −0.31; *P*=0.01; Figure 7) and medium-term (SMD = −1.20; 95% CI: −1.65 to −0.74; *P* < 0.00001). Sensitivity analysis showed that the obviously statistical heterogeneity (Chi^2^ = 12.43, df = 4; *I*^2^ = 68%) in the second subgroup was also derived from the study of Chen [46]. The high heterogeneity in the third subgroup (Chi^2^ = 7.38, df = 1; *I*^2^ = 86%) may be due to different Qigong styles. To sum up, appropriate prolonging of TCQE may be beneficial for a decrease in BP levels.

**Figure 8:**
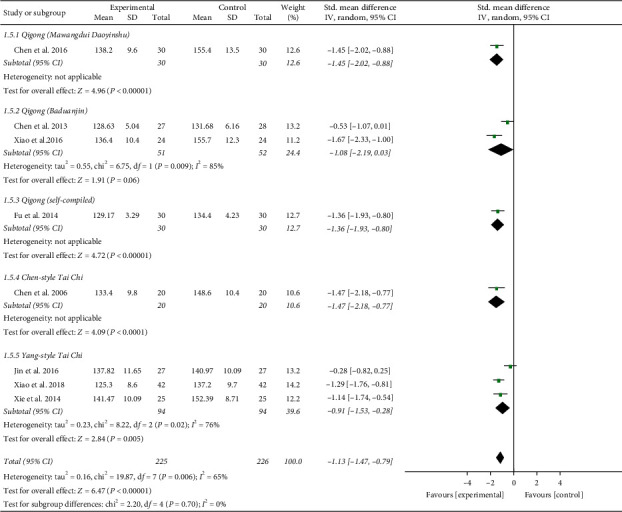
Effects of TCQE on SBP levels (subgroup based on different styles of TCQE).

**Figure 9:**
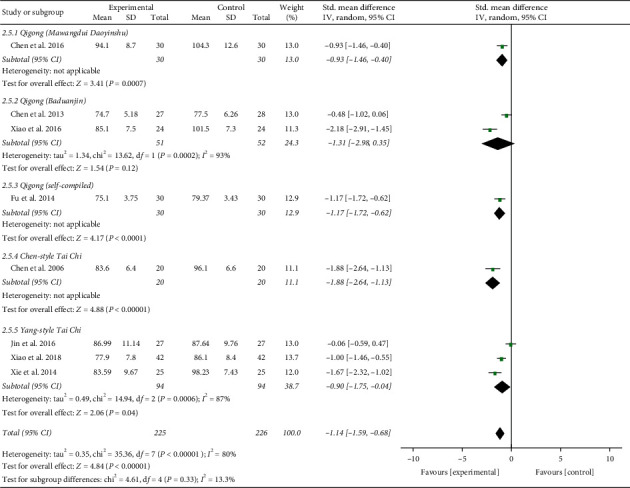
Effects of TCQE on DBP levels (subgroup based on different styles of TCQE).

Third, in the light of different styles of TCQE, we performed a subgroup analysis. Since the 8-style Tai Chi adopts the eight basic movements of Yang-style Tai Chi, we classified these two styles into the same subgroup. Because of the large *P* value (*P* ≥ 0.05) in the second subgroup, Qigong (Baduanjin) was not statistically significant in reducing BP levels. According to the size of SMD value, the effect of different TCQE on reducing SBP was arranged from big to small: Chen-style Tai Chi (SMD = −1.47; 95% CI: −2.18 to −0.77; *P* < 0.0001; Figure 8), Qigong (Mawangdui Daoyinshu) (SMD = −1.45; 95% CI: −2.02 to −0.88; *P* < 0.00001), Qigong (self-compiled) (SMD = −1.36; 95% CI: −1.93 to −0.80; *P* < 0.00001), and Yang-style Tai Chi (SMD = −0.91; 95% CI: −1.53 to −0.28; *P*=0.005). However, the results of the second (Chi^2^ = 6.75, df = 1; *I*^2^ = 85%) and fifth (Chi^2^ = 8.22, df = 2; *I*^2^ = 76%) subgroups showed statistically distinct heterogeneity. Sensitivity analysis revealed that the heterogeneity in the fifth subgroup was from the study of Jin et al. [49]. Different durations of intervention may lead to the heterogeneity in the subgroup analysis. Similarly, on the basis of SMD value, the effect of different TCQE on reducing DBP was ranked from big to small: Chen-style Tai Chi (SMD = −1.88; 95% CI: −2.64 to −1.13; *P* < 0.00001; Figure 9), Qigong (self-compiled) (SMD = −1.17; 95% CI: −1.72 to −0.62; *P* < 0.0001), Qigong (Mawangdui Daoyinshu) (SMD = −0.93; 95% CI: −1.46 to −0.40; *P*=0.0007), and Yang-style Tai Chi (SMD = −0.90; 95% CI: −1.75 to −0.04; *P*=0.04). However, the results of the second (Chi^2^ = 13.62, df = 1; *I*^2^ = 93%) and fifth (Chi^2^ = 14.94, df = 2; *I*^2^ = 87%) subgroups showed statistically distinct heterogeneity. Sensitivity analysis manifested that the heterogeneity in the fifth subgroup also came from the study of Jin et al. [49]. As mentioned above, compared with the other styles of TCQE, Chen-style Tai Chi may have the best effect on lowering BP levels, while Yang-style Tai Chi may have the least.

#### 3.3.2. Endothelial Factors

The eligible RCTs evaluated the variations of vascular endothelial function in EH patients via the concentration changes of endothelial-derived relaxing and constricting factor (NO and ET-1) before and after the interventions.

**Figure 10:**
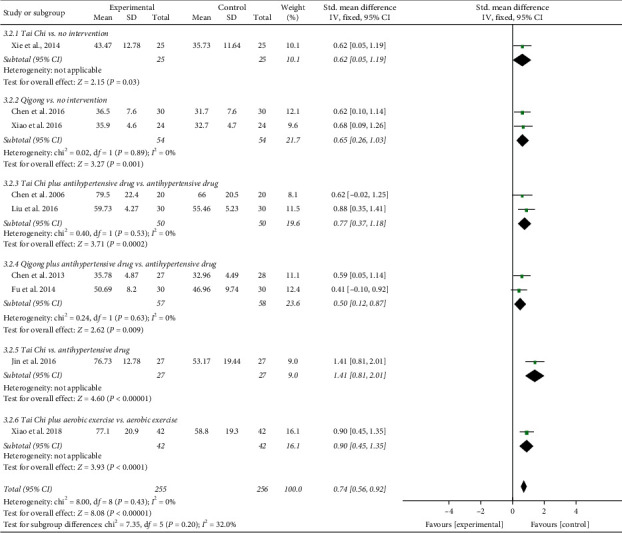
Effects of TCQE on blood levels of NO in patients with EH.

The results involving 511 EH patients of joint analysis manifested that experimental plans gave rise to more significant improvement in the NO levels compared with the control plans (SMD = 0.74; 95% CI: 0.56 to 0.92; *P* < 0.00001; fixed-effects model; Figure 10), indicating that TCQE were effective in raising the blood NO levels in patients with EH. The results of subgroup analysis also agreed with this conclusion and Tai Chi was more effective at increasing blood NO levels than antihypertensive drugs (SMD = 1.41; 95% CI: 0.81 to 2.01; *P* < 0.00001). There was no statistical heterogeneity among all studies (Chi^2^ = 8.00, df = 8; *I*^2^ = 0%).

**Figure 11:**
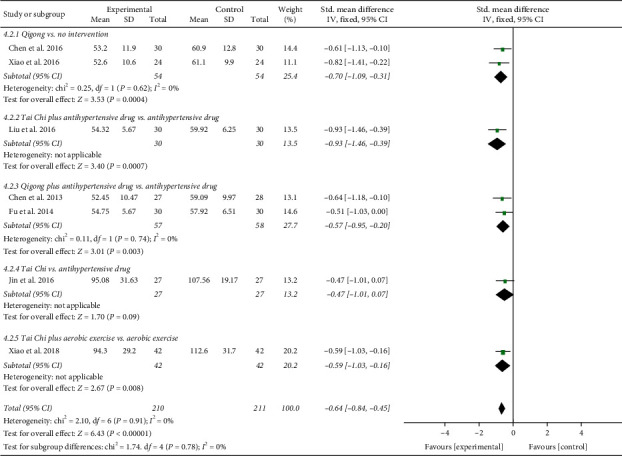
Effects of TCQE on blood levels of ET-1 in patients with EH.

In addition, the results involving 421 EH patients of both combined analysis (SMD = −0.64; 95% CI: −0.84 to −0.45; *P* < 0.00001; fixed-effects model; Figure 11) and subgroup analysis except for the fourth subgroup consistently showed that the experimental project had a greater advantage in reducing blood ET-1 levels in comparison with the control project, which primarily reflected the efficacy of TCQE in the reduction of the blood ET-1 levels for EH patients. However, according to the results of the fourth subgroup analysis, no statistically significant difference was found between Tai Chi and antihypertensive drugs in decreasing the blood ET-1 levels (SMD = −0.47; 95% CI: −1.01 to 0.07; *P*=0.09). No statistical heterogeneity existed across all trials (Chi^2^ = 2.10, df = 6; *I*^2^ = 0%).

As mentioned above, although there was no statistical significance between Tai Chi and antihypertensive drugs in regulating blood ET-1 levels of EH patients, combined result displayed that experimental options were more favorable to the regulation of vascular endothelial factor levels in the blood compared to control options, showing that TCQE could increase the blood levels of NO and decrease the blood levels of ET-1, so as to improve the endothelial function and lower BP for EH patients.

**Figure 12:**
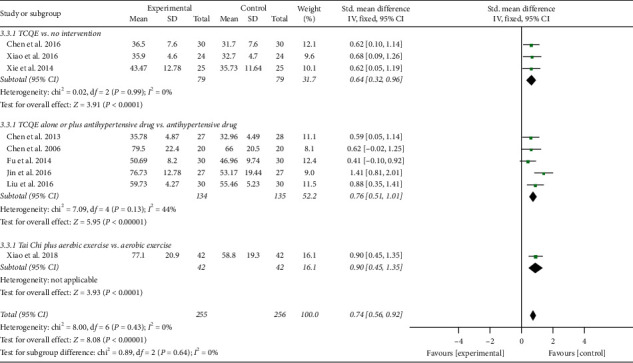
Effects of TCQE on NO levels (subgroups based on different controls).

**Figure 13:**
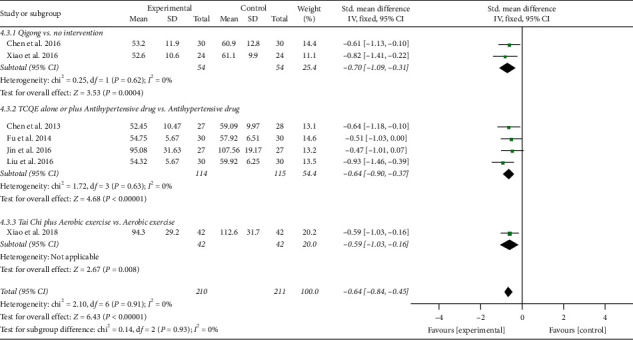
Effects of TCQE on ET-1 levels (subgroups based on different controls).

We performed further subgroup meta-analysis to explore the influence of different controls, durations of intervention or styles of exercises on the endothelial factors (NO and ET-1) in patients with EH. First, we conducted a subgroup analysis on the basis of different controls. The results demonstrated that TCQE alone or plus control plans had a better increase on NO levels when compared with control plans involving no intervention (SMD = 0.64; 95% CI: 0.32 to 0.96; *P* < 0.0001; Figure 12), antihypertensive drug (SMD = 0.76; 95% CI: 0.51 to 1.01; *P* < 0.00001), or aerobic exercise (SMD = 0.90; 95% CI: 0.45 to 1.35; *P* < 0.0001). Sensitivity analysis manifested that the statistically moderate heterogeneity (Chi^2^ = 7.09, df = 4; *I*^2^ = 44%) in the second subgroup was derived from the study of Jin et al. [49]. Different experimental plans and intervention durations may give rise to the heterogeneity. In addition, compared with control schemes, such as no intervention (SMD = −0.70; 95% CI: −1.09 to −0.31; *P*=0.0004; Figure 13), antihypertensive drug (SMD = −0.64; 95% CI: −0.90 to −0.37; *P* < 0.00001), or aerobic exercise (SMD = −0.59; 95% CI: −1.03 to −0.16; *P*=0.008), TCQE alone or plus control schemes also contributed to a better reduction of ET-1 levels. There was no statistical heterogeneity in the results of all subgroup analyses (no intervention: Chi^2^ = 0.25, df = 1; *I*^2^ = 0%; antihypertensive drug: Chi^2^ = 1.72, df = 3; *I*^2^ = 0%). In short, TCQE may be conducive to adjustment of vascular endothelial factor.

**Figure 14:**
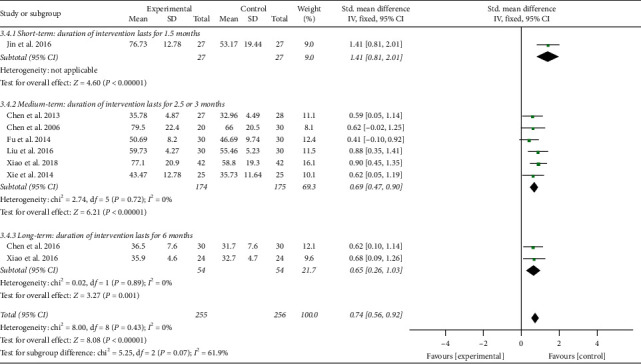
Effects of TCQE on NO levels (subgroups based on different duration of intervention).

**Figure 15:**
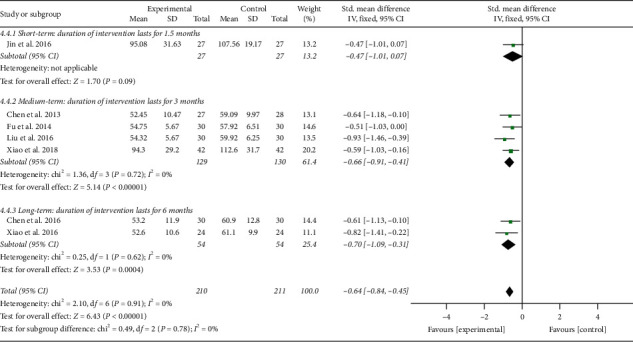
Effects of TCQE on ET-1 levels (subgroups based on different duration of intervention).

Second, based on different durations of intervention, subgroups were divided into short (1.5 months), medium (2.5 or 3 months), and long (6 months) term. Depending on the size of the SMD value, the efficacy of different intervention duration on NO levels was sorted from large to small: short-term (SMD = 1.41; 95% CI: 0.81 to 2.01; *P* < 0.00001; Figure 14), medium-term (SMD = 0.69; 95% CI: 0.47 to 0.90; *P* < 0.00001), and long-term (SMD = 0.65; 95% CI: 0.26 to 1.03; *P*=0.001). No statistical heterogeneity was found in the results of all subgroup analyses (medium-term: Chi^2^ = 2.74, df = 5; *I*^2^ = 0%; long-term: Chi^2^ = 0.02, df = 1; *I*^2^ = 0%). Furthermore, due to the large *P* value (*P* ≥ 0.05) in the first subgroup, short-term intervention had no statistical significance in reducing ET-1 levels. According to the SMD value, the effect of different intervention duration on ET-1 was also sorted from large to small: long-term (SMD = −0.70; 95% CI: −1.09 to −0.31; *P*=0.0004; Figure 15) and medium-term (SMD = −0.66; 95% CI: −0.91 to −0.41; *P* < 0.00001). The results of all subgroup analyses showed no statistical heterogeneity (medium-term: Chi^2^ = 1.36, df = 3; *I*^2^ = 0%; long-term: Chi^2^ = 0.25, df = 1; *I*^2^ = 0%). To sum up, the short-term and mid-term efficacy of TCQE may be more obvious than the long-term efficacy in increasing NO levels. However, appropriate prolonging of TCQE may be more beneficial for a decrease in ET-1 levels.

**Figure 16:**
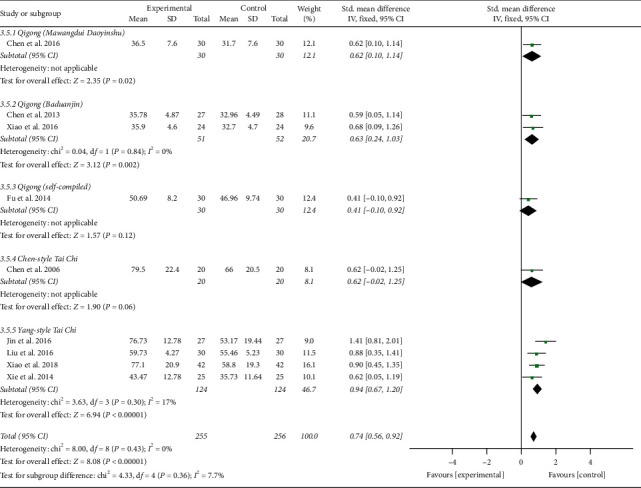
Effects of TCQE on NO levels (subgroups based on different styles of TCQE).

**Figure 17:**
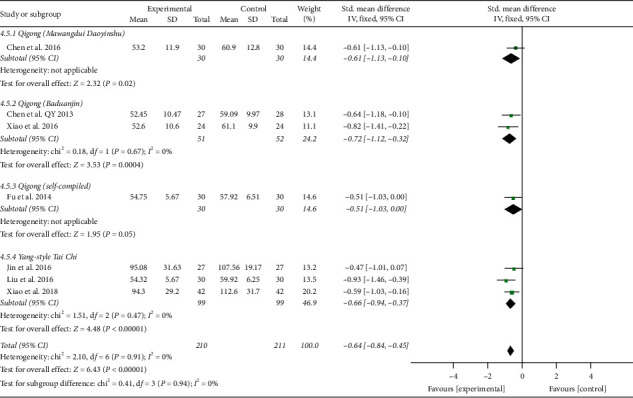
Effects of TCQE on ET-1 levels (subgroups based on different styles of TCQE).

Third, in the light of different styles of TCQE, we performed a subgroup analysis. Because of the large *P* value (*P* ≥ 0.05) in the third and fourth subgroups, Qigong (self-compiled) and Chen-style Tai Chi were not statistically significant in improving NO levels. Depending on the size of SMD value, the effect of different TCQE on improving NO was arranged from big to small: Yang-style Tai Chi (SMD = 0.94; 95% CI: 0.67 to 1.20; *P* < 0.00001; Figure 16), Qigong (Baduanjin) (SMD = 0.63; 95% CI: 0.24 to 1.03; *P*=0.002), and Qigong (Mawangdui Daoyinshu) (SMD = 0.62; 95% CI: 0.10 to 1.14; *P*=0.02). Sensitivity analysis revealed that the statistically low heterogeneity (Chi^2^ = 3.63, df = 3; *I*^2^ = 17%) in the fifth subgroup was from the study of Jin et al. [49], revealing that different intervention durations may lead to heterogeneity. Similarly, Qigong (self-compiled) was not statistically significant in lowering ET-1 levels. On the basis of SMD value, the effect of different TCQE on reducing ET-1 was ranked from big to small: Qigong (Baduanjin) (SMD = −0.72; 95% CI: −1.12 to −0.32; *P*=0.0004; Figure 17), Yang-style Tai Chi (SMD = −0.66; 95% CI: −0.94 to −0.37; *P* < 0.00001), and Qigong (Mawangdui Daoyinshu) (SMD = −0.61; 95% CI: −1.13 to −0.10; *P*=0.02). We found no statistical heterogeneity in the results of all subgroup analyses (Qigong [Baduanjin]: Chi^2^ = 0.18, df = 1; *I*^2^ = 0%; Yang-style Tai Chi: Chi^2^ = 1.51, df = 2; *I*^2^ = 0%). As described above, compared with other styles of TCQE, Yang-style Tai Chi and Qigong (Baduanjin) may have more obvious effect on adjustment of vascular endothelial factor levels in EH patients, while Qigong (Mawangdui Daoyinshu) may have less.

### 3.4. Methodological Quality of Included Studies

**Figure 18:**
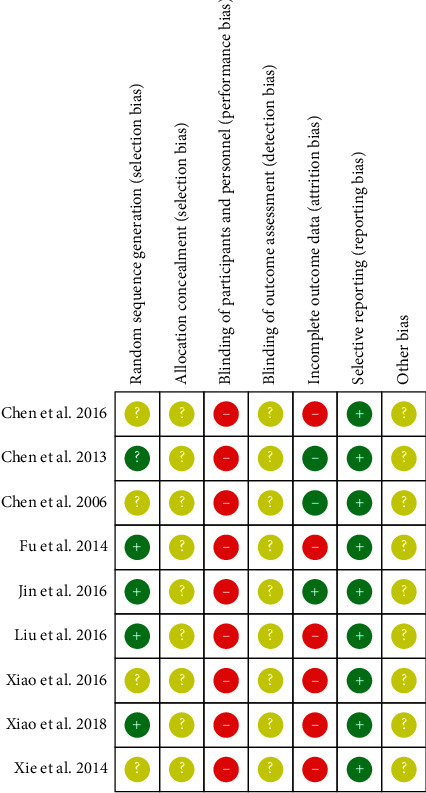
Risk of Bias in the included RCTs (red, yellow, and green labels indicate high risk, unclear risk, and low risk, respectively).

According to The Cochrane Risk of Bias, the overall methodological quality of the included RCTs was evaluated as low and presented in Figure 18. All RCTs mentioned randomization, while five of them illustrated the methods of random sequence generation, which was by “random number table” [46, 48, 49, 52]; one of them divided participants into experimental groups and control groups by “randomization software” (excel software) [50]. None of the RCTs described details about allocation concealment or assessor blinding, which was assessed as an unclear risk. It is generally impossible to blind participants and personnel in RTCs of TCQE interventions, so a high risk of performance bias existed in all trials. Since information about dropouts was not reported, a high risk of attrition bias could not be ruled out in six trials [45, 48, 50–53]. All trials recorded all outcomes mentioned in their methods section, which had a low risk of reporting bias. All studies were assessed as an unclear risk of other bias due to lack of necessary interpretable information.

## 4. Discussion

HT affects over 1.2 billion individuals worldwide and has become the most critical and expensive public health problem, accounting for around 10% of worldwide healthcare costs [4, 56]. Only 24% of hypertensive patients have their HT under control [57]. Effectiveness of antihypertensive medications is limited by noncompliance, availability, high costs, and negative side effects in mood state, cognitive functioning, and sexual performance [18, 22, 58]. Therefore, a growing body of research in the West has focused upon lifestyle modification as an alternative to hypotensive drugs. However, there is insufficient evidence to suggest that lifestyle modifications alone reduce morbidity or mortality in hypertensive patients [22, 57]. The importance of regular physical activity in EH has been extensively investigated over the last decades and has emerged as a major modifiable factor contributing to optimal BP control [59]. However, cost-effectiveness of therapy becomes an important consideration [60]. Traditional exercise studies focus on laboratory training requiring expensive equipment. Although a high-technology programme is effective in short-term training, practising it in everyday life is difficult [61].

TCQE is promoted as a viable, affordable, accessible alternative [38, 62]. Furthermore, there is little or no potential harm in doing these slow-dance exercises from China [63]. With reputed health benefits, this form of physical activity has apparent safety for people of all ages, including older adults and medically compromised populations, and irrespective of previous exercise experience [24]. For finding the relevant testimony of TCQE in making decisions for EH patients, numerous clinical RCTs have been performed to enhance credibility [38]. It is significative to summary and analysis of the current clinical RCTs of evidence-based TCQE for EH patients. Therefore, we conducted meta-analysis to objectively evaluate all the qualified RCTs and investigated the potential efficacy of TCQE for EH. However, it is still unclear if the decline in BP from TCQE is due to exercise-related changes in levels of endothelial-derived relaxing factor and constricting factor.

During arterial hypertension, while NO release in response to hemodynamic stress is downregulated, the synthesis of angiotensin-converting enzyme and powerful vasoconstrictor ET-1 is increased [64]. Normally, there is a balance between vasoconstrictive and vasodilating substances in the vasculature but in HT, the bioavailability of endothelin might be increased in parallel with a reduction in NO bioactivity [5]. HT is characterized by a decline in endothelial function [65]. Reversal of endothelial function, by both nonpharmacological methods and antihypertensive medications and therapies, is more directly targeted at the endothelium [5]. Although ED is a conceptually attractive therapeutic target in HT, at this point in time, we lack convincing data that using ED to guide our treatment would produce any better outcomes than using blood pressure targets to guide our treatment. This situation may change in the near future as more trials are conducted in this area [5]. The clinical RCTs testing the effectiveness of TCQE on improvement of vascular endothelial function in EH patients have never been systematically summarized. Hence, in this review, we assumed that NO and ET-1 were indices of vascular endothelial function and conducted the meta-analysis. Our study also aimed to evaluate the role of endothelial-derived factors (NO and ET-1) in the reduced BP associated with TCQE. Future therapy for HT must be directed at not only lowering blood pressure but also reversing structural alterations in the vasculature [66]. Investigation of the relationship linking ED and EH, as well as assessment of the effectiveness of TCQE targeting endothelial-derived factor in EH patients seem to be of great significance.

Although Tai Chi has no obviously statistical advantage in lowering BP and blood ET-1 levels of EH patients compared with antihypertensive drugs, the results of comprehensive analysis showed that after 1.5 to 6 months of intervention, compared with control schedules, experimental schedules were more effective in reducing the BP and contributed higher blood levels of NO and lower blood levels of ET-1 for EH patients. This meta-analysis indicated that TCQE could regulate the levels of endothelial-derived factors (NO and ET-1) that are associated with an improved vascular endothelial function, which in turn can be favorable factors for demonstrated positive effects of TCQE on EH. Besides, the data of our meta-analysis revealed that the variations in SBP and DBP were negatively correlated with the variations in NO and positively correlated with the variations in ET-1. Changes in blood levels of these endothelial-derived relaxing factor and constricting factor may improve the function of endothelium-associated vasodilation and contraction in patients with EH. Hence, Tai Chi and Qigong exercise-related increases in NO levels and decreases in ET-1 levels may be potential mechanisms for lowering BP in EH patients. Furthermore, we reported subgroup analysis based on different controls, durations of intervention, or styles of TCQE. Firstly, the results manifested that compared with control plans including no intervention, antihypertensive drug, or aerobic exercise, TCQE alone or plus control plans made a contribution to a better reduction of BP and ET-1 levels, and a better improvement of NO levels. Secondly, although there is no evidence that the effect of TCQE for NO levels is better in the long-term than in the short-term and mid-term, appropriate prolonging of TCQE could be more beneficial for a decrease in BP and ET-1 levels. Thirdly, compared with the other styles of TCQE, Chen-style Tai Chi may have better effect on lowering BP levels, while Yang-style Tai Chi may have worse. However, Yang-style Tai Chi and Qigong (Baduanjin) may have more obvious efficacy on adjustment of vascular endothelial factor levels, while Qigong (Mawangdui Daoyinshu) may have less. The antihypertensive effect of different TCQE styles was not completely consistent with their effect on regulating levels of vascular endothelial factor. The reason may be that the small number of RCTs included and some variables among different RCTs affected the accurately comparative results of different TCQE styles in changing the levels of BP and endothelial factors.

We were only able to make very preliminary conclusions based on small volume of data. The following limitations should be considered in our meta-analysis:

1. The methodological quality of the included RCTs was generally low on the basis of Cochrane Risk of Bias. Randomization was reported in nearly half of the included trials but with no further details. The majority of RCTs provided inadequate information of allocation concealment. It was very difficult to design blinding of participants in clinical RCTs of TCQE intervention. This difficulty was largely overcome by blinding of outcome assessment. However, the primary defect in all the included trials was the lack of information about blinding those analyzing the results. Information about dropouts was inadequate in the majority of trials. Thus, potential “high” or “unclear” Risk of Bias might be generated in most of the included RCTs.
2. The so-called “legacy effect” in the treatment of HT, in which patients who are treated with a given antihypertensive therapy may derive a long-term benefit after discontinuation of therapy, has been recently proposed on the basis of accumulating evidence and, in particular, on the availability of long-term posttrial observations in randomized controlled clinical trials [16]. Trial lengths of 1.5 to 6 months and lack of long-term follow-up in all of the included RCTs might not be sufficient to investigate the legacy effect and safety of TCQE for EH.
3. Each included study was single centre and relatively small scale RCTs.
4. Although we carried out comprehensive and unbiased data retrieval as much as possible, all of the finally qualified trials were performed in China and most of them were published in China. Because journals from Asian countries are more likely to publish positive results favoring TCQE, there may be potential publication bias.
5. We only conducted a retrieval for Chinese and English RCTs from seven databases, which may well cause a selection bias.
6. In the trials, a number of differences existed in the interventions of the control groups, especially the regimens of antihypertensive drugs.
7. Due to heterogenous characteristics of participants, different styles of TCQE, differences in frequency and duration of the training, and variational tools of outcome measurement, the wide clinical heterogeneity of the included RCTs was also a limitation in this analysis.
8. Detailed information of adverse effects related to TCQE practices was ignored in the included trials.
9. None of the included trials reported methods of evaluating vascular function, such as ultrasound flow-mediated vasodilation, angiography with acetylcholine injection, or reactive hyperemia index, so it was difficult for us to further examine the influence of TCQE on vascular function in EH patients.

The positive clinical evidence of TCQE for EH patients should be interpreted prudently because of the above numerous limitations and deficiencies in this meta-analysis. Despite of the fact that the meta-analysis of current RCTs is hopeful as regards the effect in regulating levels of endothelial-derived factors to lower BP for EH patients via TCQE, additional large-scale, longer, more rigorous studies are required to prove the effectiveness of TCQE in treatment strategy of endothelial function in EH patients and the exact mechanism of lowering BP of EH patients in TCQE.

The potential efficiency for reducing BP by improving endothelial function, the low cost, and the safety, point to the importance of promoting TCQE to improve the lives and health of the patients with EH. Perhaps in the future, with the progress in research methods and the increase of favorable evidence, TCQE will be evaluated as health-friendly interventions incorporated into community, nursing homes, or hospital and play an important part in the emerging integrative medicine system. To achieve this goal and provide necessary evidence for future clinical trials, a number of confusions may need to be resolved. TCQE include different styles, teachers, lengths, and frequencies. Due to the numerous deficiencies in our meta-analysis, the exciting evidence and credibility of conclusion are limited. It is not entirely certain whether one style might be better for some conditions than others, if longer classes are better than shorter classes or whether 2 or more classes a week is optimal. Although it is useful to know that benefits can be seen after only a few weeks, those who have practised Tai Chi for many years would note that benefits continue to accrue even after decades of practice [27]. It is clear that future researches should pay attention to confirm the characteristics of EH patients most likely to benefit from TCQE, the most suitable frequency and duration of exercises, and the most effective styles of TCQE.

## 5. Conclusion

In conclusion, TCQE are an effective therapy for EH. The lower BP in EH patients who practice TCQE may have some connection with exercise-related variations in blood levels of endothelial-derived relaxing and constricting factor, such as endogenous NO and ET-1, which take part in the regulation of vascular endothelial function. Our meta-analysis also supplies initial evidence-based medical evidence for making a probable use of TCQE as a complementary and alternative therapy in EH patients. However, because of the generally low methodological quality of the included RCTs, the exciting evidence and credibility of conclusion are limited. Rigorously designed, larger scale and long-term follow-up RCTs are required to confirm the efficacy of TCQE in management of EH and further research is needed to make clear the mechanism of lowering BP in TCQE. Perhaps in the future, based on a wealth of reliable medical evidence, TCQE may be recommended as a complementary and alternative therapy for the effective treatment of global EH.

## Figures and Tables

**Table 1:** Electronic search strategies.

Database	Search terms
Cochrane	#1 MeSH descriptor: [Tai Ji] explode all trees
	#2 MeSH descriptor: [Qigong] explode all trees
	#3 MeSH descriptor: [Hypertension] explode all trees
	#4 (Tai-ji): ti, ab, kw
	#5 (Tai Chi): ti, ab, kw
	#6 (Tai Chi Chuan): ti, ab, kw
	#7 (Tai Ji Quan): ti, ab, kw
	#8 (Taiji): ti, ab, kw
	#9 (Taijiquan): ti, ab, kw
	#10 (Qi Gong): ti, ab, kw
	#11 (Qi-Gong): ti, ab, kw
	#12 (qi gong): ti, ab, kw
	#13 (High Blood Pressure): ti, ab, kw
	#14 (High Blood Pressures): ti, ab, kw
	#15 #1 OR #4 OR #5 OR #6 OR #7 OR #8 OR #9
	#16 #2 OR #10 OR #11 OR #12
	#17 #3 OR #13 OR #14
	#18 #15 OR #16
	#19 #17 AND #18

PubMed	1.“Hypertension”[Mesh] OR High Blood Pressures [Title/Abstract] OR Blood Pressure, High [Title/Abstract] OR Blood Pressures, High [Title/Abstract] OR High Blood Pressure [Title/Abstract]
	2. “Tai Ji” [Mesh] OR Tai Chi Chuan [Title/Abstract] OR Tai-ji [Title/Abstract] OR Tai Chi [Title/Abstract] OR Chi, Tai [Title/Abstract] OR Tai Ji Quan [Title/Abstract] OR Ji Quan, Tai [Title/Abstract] OR Quan, Tai Ji [Title/Abstract] OR Taiji [Title/Abstract] OR Taijiquan [Title/Abstract] OR T'ai Chi [Title/Abstract]
	3. “Qigong” [Mesh] OR Ch'i Kung [Title/Abstract] OR Qi Gong [Title/Abstract]
	4. randomized controlled trial [Publication Type] OR randomized [Title/Abstract] OR placebo [Title/Abstract]
	5. 2 OR 3
	6. 1 AND 4 AND 5

Embase	#1 ‘Tai Chi'/exp
	#2 ‘tai ji': ab, ti
	#3 ‘tai chi chuan': ab, ti
	#4 ‘tai-ji': ab, ti
	#5 ‘tai ji quan': ab, ti
	#6 ‘taiji': ab, ti
	#7 ‘taijiquan': ab, ti
	#8 ‘qi gong'/exp
	#9 ‘qigong': ab, ti
	#10 ‘hypertension'/exp
	#11 ‘high blood pressure': ab, ti
	#12 ‘high blood pressures': ab, ti
	#13 ‘randomized controlled trial'/exp
	#14 #1 OR #2 OR #3 OR #4 OR #5 OR #6 OR #7
	#15 #8 OR #9
	#16 #10 OR #11 OR #12
	#17 #14 OR #15
	#18 #13 AND #16 AND #17

CNKI	1. Central-Theme: tai ji (Tai Chi)
	2. Central-Theme: qi gong (Qigong) or ba duan jin (Baduanjin) or yi jin jing (Yijinjing) or wu qin xi (Wuqinxi)
	3. 1 or 2
	4. Central-Theme: gao xue ya (hypertension)
	5. 3 and 4
	6. Title/Keywords/Abstract: tai ji (Tai Chi)
	7. Title/Keywords/Abstract: qi gong (Qigong) or ba duan jin (Baduanjin) or yi jin jing (Yijinjing) or wu qin xi (Wuqinxi)
	8. 6 or 7
	9. Title/Keywords/Abstract: gao xue ya (hypertension)
	10. 8 and 9
	11. 5 or 10

VIP	1. Title/Keywords: tai ji (Tai Chi)
	2. Title/Keywords: qi gong (Qigong) or ba duan jin (Baduanjin) or yi jin jing (Yijinjing) or wu qin xi (Wuqinxi)
	3. 1 or 2
	4. Title/Keywords: gao xue ya (hypertension)
	5. 3 and 4
	6. Abstract: tai ji (Tai Chi)
	7. Abstract: qi gong (Qigong) or ba duan jin (Baduanjin) or yi jin jing (Yijinjing) or wu qin xi (Wuqinxi)
	8. 6 or 7
	9. Abstract: gao xue ya (hypertension)
	10. 8 and 9
	11. 5 or 10

Wanfang data	1. Subject: tai ji (Tai Chi)
	2. Subject: qi gong (Qigong) or ba duan jin (Baduanjin) or yi jin jing (Yijinjing) or wu qin xi (Wuqinxi)
	3. 1 or 2
	4. Subject: gao xue ya (hypertension)
	5. 3 and 4
	6. Title/Keywords: tai ji (Tai Chi)
	7. Title/Keywords: qi gong (Qigong) or ba duan jin (Baduanjin) or yi jin jing (Yijinjing) or wu qin xi (Wuqinxi)
	8. 6 or 7
	9. Title/Keywords: gao xue ya (hypertension)
	10. 8 and 9
	11. Abstract: tai ji (Tai Chi)
	12. Abstract: qi gong (Qigong) or ba duan jin (Baduanjin) or yi jin jing (Yijinjing) or wu qin xi (Wuqinxi)
	13. 11 or 12
	14. Abstract: gao xue ya (hypertension)
	15. 13 and 14
	16. 5 or 10 or 15

CBM	1. Title: tai ji (Tai Chi)
	2. Title: qi gong (Qigong) or ba duan jin (Baduanjin) or yi jin jing (Yijinjing) or wu qin xi (Wuqinxi)
	3. 1 or 2
	4. Title: gao xue ya (hypertension)
	5. 3 and 4
	6. Abstract: tai ji (Tai Chi)
	7. Abstract: qi gong (Qigong) or ba duan jin (Baduanjin) or yi jin jing (Yijinjing) or wu qin xi (Wuqinxi)
	8. 6 or 7
	9. Abstract: gao xue ya (hypertension)
	10. 8 and 9
	11. 5 or 10

**Table 2:** Characteristics of included studies.

Study ID	Sample (E/C)	Age (years)	Sex (M/F)	Diagnosis standard	Hypertension classification (E/C)	Frequency	Experimental groups	Control groups	Duration	Outcome indicators	Dropout (E/C)
Chen [45]	30/30	66.3 ± 5.8	NR	NR	EH	44 min/day, 5 days/week	Qigong (Mawangdui Daoyinshu)	No intervention	6 months	SBP, DBP, NO, ET-1	NR
Chen [46]	30/30	E: 69.23 ± 3.72 C: 70.06 ± 3.51	E: 13/14 C: 16/12	GPTHC (2005 edition)	EH, stage I (60)	30 min/day, 5 days/week	Qigong (Baduanjin) + antihypertensive drug	Antihypertensive drug (amlodipine besylate 5 mg qd or telmisartan 80 mg qd)	3 months	SBP, DBP, NO, ET-1	3/2
Chen [47]	20/20	E: 64.3 C: 60.7	E: 9/11 C: 13/7	GPTHC (2004 edition)	EH, stage II (26), stage III (14)	40 min/day, 7 days/week	Chen-style Tai Chi + antihypertensive drug	Antihypertensive drug (nifedipine sustained-release tablets, EH of stage II: 25 mg bid; EH of stage III: 50 mg bid)	2.5 months	SBP, DBP, NO	0/0
Fu [48]	30/30	E: 57.93 ± 6.63 C: 59.53 ± 7.46	E: 20/10 C: 19/11	GPTHC (2010 edition)	EH, stage I (24/21), stage II (6/9)	40 min/day, 6 days/week	Qigong (self-compiled) + antihypertensive drug	Antihypertensive drug (CCB, ACEI, ARB, DAD. NR specific drug and dosage)	3 months	SBP, DBP, NO, ET-1	NR
Jin et al. [49]	27/27	56.9 ± 5.7	11/43	GPTHC (2010 edition)	EH, stage I (54)	40 min/day, 7 days/week	24-movement Yang-style Tai Chi	Antihypertensive drug (amlodipine 2.5 mg qd)	1.5 months	SBP, DBP, NO, ET-1	0/0
Liu [50]	30/30	E: 56.8 ± 6.78 C: 56.33 ± 7.16	E: 16/14 C: 19/11	GPTHC (2015 edition)	EH, stage I (19/21), stage II (11/9)	40 min/day, 5 days/week	24-movement Yang-style Tai Chi + antihypertensive drug	Antihypertensive drug (amlodipine besylate 5 mg qd)	3 months	NO, ET-1	NR
Xiao et al. [51]	24/24	65.6 ± 7.8	NR	NR	EH	40 min/day, 5 days/week	Qigong (Baduanjin)	No intervention	6 months	SBP, DBP, NO, ET-1	NR
Xiao [52]	42/42	E:60.2 ± 4.6 C: 60.5 ± 4.9	E: 24/18 C: 22/20	GPTHC (2010 edition)	EH	60 min/day, 5 days/week	8-style Tai Chi + aerobic exercise	Aerobic exercise (jog and walk fast)	3 months	SBP, DBP, NO, ET-1	NR
Xie et al. [53]	25/25	60–70	E: 11/14 C: 14/11	GPTHC (2005 edition)	EH, stage I (20), stage II (30)	60 min/day, 5 days/week	24-movement Yang-style Tai Chi	No intervention	3 months	SBP, DBP, NO	NR

E: experimental group, C: control group, M: male, F: female, EH: essential hypertension; NR: not reported; GPTHC: Guidelines for Prevention and Treatment of Hypertension in China, SBP: systolic blood pressure, DBP: diastolic blood pressure, NO: nitric oxide, ET-1: endothelin-1, CCB: calcium channel blockers, ACEI: angiotensin-converting enzyme inhibitor, ARB: angiotensin receptor blocker, DAD: diuretic antihypertensive drug, qd: quaque die/once day, bid: bis in die/twice day. Diagnosis standard: GPTHC (2004 edition): phase II (160 mmHg ≤ SBP ≤ 179 mmHg and/or 100 mmHg ≤ DBP ≤ 109 mmHg), phase III (SBP ≥ 180 mmHg and/or DBP ≥ 110 mmHg). GPTHC (2005 edition): phase I (140 mmHg ≤ SBP ≤ 159 mmHg, and/or 90 mmHg ≤ DBP ≤ 99 mmHg), phase II (160 mmHg≤ SBP < 180 mmHg and/or 100 mmHg ≤ DBP < 110 mmHg). GPTHC (2010 edition): phase I (140 mmHg ≤ SBP ≤ 159 mmHg and/or 90 mmHg ≤ DBP ≤ 99 mmHg), phase II (160 mmHg ≤ SBP < 179 mmHg and/or 100 mmHg ≤ DBP < 109 mmHg). GPTHC (2015 edition): phase I (140 mmHg ≤ SBP ≤ 159 mmHg and/or 90 mmHg ≤ DBP ≤ 99 mmHg), phase II (160 mmHg ≤ SBP ≤ 179 mmHg and/or 100 mmHg ≤ DBP ≤ 109 mmHg). Additional description: the 8-style Tai Chi is also called Yi-duan Quan and adopts the eight main and basic movements of Yang-style Tai Chi. There are 10 movements in the 8-style Tai Chi (including starting and ending). The exercise moves from slow to slightly fast and then from slightly fast to slowly end. Chen-style Tai Chi is characterized by the combination of Yin and Yang and the combination of hardness and softness. This exercise has slow/soft movements similar to the Yang-style and fast/hard movements similar to aerobic exercises.

## Data Availability

The data and materials used in this study are included within the article.

## Abbreviations

TCQE: Tai Chi and Qigong exercises
RCT(s): Randomized controlled trial(s)
HT: Hypertension
EH: Essential hypertension
BP: Blood pressure
SBP: Systolic blood pressure
DBP: Diastolic blood pressure
NO: Nitric oxide
ET-1: Endothelin-1
ED: Endothelial dysfunction
TCM: Traditional Chinese medicine
SMD: Standardized mean difference
CI: Confidence interval
Chi^2^: Chi-square
df: Degree of freedom.
